# The Association Between Persisting Symptoms after Concussion (PSaC) and Symptoms of Depression and/or Anxiety in the Athletic Population: A Scoping Review

**DOI:** 10.17159/2078-516X/2026/v38i1a24812

**Published:** 2026-06-15

**Authors:** MJ Lumb, KE Welman, N Snegireva

**Affiliations:** Department of Exercise, Sport & Lifestyle Medicine, Division of Movement Science and Exercise Therapy, The Movement Laboratory, Faculty of Medicine and Health Sciences, Stellenbosch University, South Africa

**Keywords:** mental health, screening tools, sport-related concussion, athletes, persistent post-concussion symptoms

## Abstract

**Background:**

The association between persisting symptoms after concussion (PSaC) and symptoms of depression and/or anxiety is a growing concern in athlete populations. However, the evidence base is complex and inconsistent, creating significant diagnostic challenges and uncertainty regarding appropriate screening protocols. This scoping review maps the literature from the past five years to examine the evidence for this association in adult athletes and to identify the screening tools employed.

**Objectives:**

The objectives of this scoping review were: (1) to systematically map the extent, range, and nature of the empirical literature on the association between PSaC and symptoms of depression and/or anxiety in adult athletic populations; (2) to identify and categorize the screening tools used to assess symptoms of depression and/or anxiety; and (3) to identify gaps and limitations in the existing body of research to provide direction for future investigations. This review was guided by the following questions: 1) What is the nature of the reported association between PSaC and symptoms of depression and/or anxiety in adult athletes? 2) What screening tools are most frequently utilized in the literature to assess symptoms of depression and/or anxiety in this population?

**Methods:**

This scoping review followed the Joanna Briggs Institute (JBI) methodology, incorporating the framework by Arksey and O’Malley and subsequent JBI refinements. A systematic search of English-language literature was conducted in the PubMed, Scopus, and Google Scholar databases. Studies were included if they: (1) involved adult populations (≥18 years); (2) focused on sport-related concussion (SRC) as a mechanism of injury; (3) assessed symptoms persisting for four weeks or more (i.e., PSaC); and (4) explored the association between PSaC and symptoms of depression and/or anxiety.

**Results:**

The review identified 14 studies. A majority (n=9) reported a positive association between PSaC and symptoms of depression and anxiety. However, the overall evidence base was marked by significant methodological heterogeneity, including a wide range of study designs, participant numbers (ranging from single case reports to large surveys (n = 1–3406), populations (from active to retired athletes), and time since injury (28 days to 55 years). There was a lack of consensus in assessment methods, with an inconsistent collection of 13 different screening tools used across the included studies to measure symptoms of depression and/or anxiety. Furthermore, a foundational challenge identified was the inconsistent terminology used across studies to describe persisting symptoms.

**Conclusion:**

While the evidence suggests a link between PSaC and symptoms of depression and/or anxiety in athletes, a lack of consensus on terminology and screening tools severely limits the comparability of research and the development of clear clinical guidelines. This highlights a potential need for standardisation to improve the quality of research and, ultimately, the clinical care and well-being of athletes with PSaC.

Persistent symptoms after concussion (PSaC) are a pathological manifestation of symptoms that persist after the initial clinical signs of concussion are expected to subside.^[1,2]^ It has been defined by numerous terms, including post-concussion syndrome (PCS) and persistent post-concussive or post-concussion symptoms (PPCS).^[1]^ Recently, experts have suggested that the term PSaC should be used, as it emphasises that the symptoms are related to the initial injury and are treatable, even when present beyond the typical timeframe.^[1–3]^ This gives patients and practitioners an opportunity to discuss a clear, active treatment plan that focuses on recovery.

Sport-related concussions (SRCs) are unique in nature when compared to other mechanisms of head injury due to such factors as repetitive sub-concussive impacts (i.e. accumulated load), pressure placed on the athlete to return to play as soon as possible, the stigma surrounding head impact injuries, as well as the impact SRCs could have on athletic identity.^[2–4]^ Athletes may also experience increased psychological stressors due to performance expectations placed on themselves and by others, as well as fear of re-injury.^[2,3]^ These may result in exacerbation of symptoms of depression and/or anxiety, which may persist for weeks.^[2,3]^ These factors are unique to SRCs and highlight differences when compared to other mechanisms of concussion.^[2,3]^ Therefore, homogeneity in samples is essential for yielding clearer, more reliable results in the study of these complex pathologies.

PSaC is generally conceptualised within three symptom domains, including physical (headaches, dizziness, blurred vision, etc.), cognitive (memory and attention difficulties, etc.), and emotional (irritability, anxiousness, depression, etc.).^[1–3]^ For the purpose of this review, symptoms of anxiety and depression were selected for investigation as they are the most frequently assessed emotional outcomes in PSaC literature and are symptoms most consistently associated with prolonged recovery in the athletic population.^[3,5–7]^ In this review, symptoms of depression and/or anxiety were defined as outcomes operationalised via validated screening instruments (e.g., PHQ-9, GAD-7, HADS subscales, etc.) and treated as separate constructs, consistent with how they were assessed and reported across included studies (Supplementary Table 3).

Previous reviews have noted that depression and anxiety are primarily focused on in SRC literature, further noting that other emotional outcomes have had minimal research conducted on them.^[5,7]^ The prioritisation of symptoms of depression and anxiety in this review supports the unique psychological paradigm of the athletic population mindset, where performance expectations, return to play pressure, and the negative connotations athletes potentially face surrounding head injuries may predispose them to increased presentations of these specific symptoms.^[2,3,6]^

Athletes also potentially present with unique cognitive discrepancies following head injuries, particularly the contrast between their self-perceived capabilities prior to suffering a concussion and their current state of being. This self-perceived discrepancy presents a direct threat to the athletic population that has been associated with symptoms of depression and apathy specifically.^[3,8]^

It has been suggested that PSaC is present in about 10–35% of athletes, depending on the cohort and the time frames used to define persisting symptom presentation.^[6,9,10]^ This prevalence underscores the clinical significance of the questions addressed in this review.

Symptoms of depression and/or anxiety have been associated with PSaC within a mixed population; however, the evidence is still inconclusive due to the methodological variations, lack of longitudinal data, and, in part, conflicting results, especially when exploring these associations in homogenous groups.^[3,5,11]^

If symptoms of depression and/or anxiety in athletes go undiagnosed and untreated, a potential for further psychological manifestations may present.^[5,7]^ This in turn may result in an increased risk of physiological impairment and of musculoskeletal injuries, both of which have the potential to impact an individual’s quality of life and sports performance, potentially leading to further mental health degradation.^[3,12,13]^

A bidirectional relationship appears to exist between concussions and symptoms of depression and/or anxiety. On one hand, athletes with pre-existing depressive and anxiety symptoms are more than twice as likely to sustain future concussions, suggesting these symptoms are unique risk factors that should be screened for.^[14,15]^ Conversely, increases in the number of concussion incidents also correlate with increases in symptoms of depression and/or anxiety, pointing to a brain-body connection that requires further exploration.^[10]^ In addition, pre- and post-concussive depression and anxiety may similarly affect the athletes’ return to play. However, predictive factors are not conclusive as of yet and further research is required to standardise symptoms of depression and/or anxiety screening within this specific population.^[14]^

The relationship between post-concussion symptoms and mental health has been a subject of recent reviews; however, none explored associations between PSaC and symptoms of depression and/or anxiety specifically in athletes.^[5,7,16,17]^ The most notable review, by Lambert et al. (2022), confirmed a link between PPCS and a risk of experiencing depressive symptoms.^[5]^ While foundational, this work highlights the need for a more specific focus, as it was limited solely to depressive outcomes, used outdated terminology and a mixed population. Further research has reinforced the complexity of the relationship between PPCS and symptoms of depression and/or anxiety. A systematic review by Iverson et al. (2020) established that pre-injury mental health problems exacerbate clinical outcomes following a sport-related concussion (SRC), highlighting a need to stratify these risks in specific populations and develop targeted rehabilitation protocols.^[16]^ Complementing this, a meta-analysis by Langdon et al. (2020) identified at least five distinct subtypes of SRC symptomatology, including cognitive-emotional and sleep-emotional clusters, demonstrating that post-concussion pathways are varied rather than uniform.^[17]^

It was concluded that identifying these separate clusters may enable individualised treatment and management based on subtype, with the goal of improving patient clinical outcomes.^[17]^ Finally, a review by Sheldrake et al. (2022) investigated mental health outcomes across paediatric and adult populations with persistent post-concussion symptoms. The authors highlighted the importance of a holistic approach in clinical and research settings that addresses both the physical and mental health needs of individuals with PPCS.^[7]^

Thus, the research on post-concussion mental health in athletes is marked by significant inconsistencies, and no recent review has specifically examined the definitions, populations, and assessment methods for PSaC. Therefore, this scoping review set out to examine the relevant literature from the past five years to comprehensively map the existing evidence, clarify key concepts, and identify distinct gaps for future investigation. This scoping review sets the foundation for improved methodological coherence on this nuanced topic, supporting the design of future hypothesis-driven studies, including systematic reviews with meta-analysis. The five-year scope of this review was deliberately chosen to capture a period of rapid evolution, anchored by the 2023 Consensus Statement on concussion in sport.^[1–3]^ This ensures a synthesis of the critical research published both before and after the Statement, providing a current analysis to identify the most relevant gaps in the literature.

South Africa’s high levels of participation in contact sports across school, university, club, and national settings, particularly in rugby, make the identification and management of PSaC directly relevant to local health practitioners. At the same time, the heterogeneity of the studies and the findings identified in this review closely reflect patterns reported in the international concussion literature, supporting the broader applicability of these findings beyond the local context.

## Methods

### Review design and registration

The scoping review followed the JBI methodology for scoping reviews, incorporated elements from the framework of Arksey and O’Malley and took into consideration refinements suggested by the Joanna Briggs Institute.^[18–21]^ Literature search took place between October 2024 and March 2025. The scoping review was registered on the Open Science Framework (OSF) platform to provide transparency and accountability, and to help reduce bias and duplication on the topic, as recommended by the Preferred Reporting Items for Systematic Reviews and Meta-Analyses extension for Scoping Reviews (PRISMA-ScR).^[22]^ Details regarding the search strings used can be found in Supplementary Table 1.

### Search strategy and database selection

Three databases were scoped: Google Scholar, PubMed, and SCOPUS. Inclusion criteria were 1) empirical studies only (thus excluding editorials, opinion pieces, etc.), 2) only published manuscripts and dissertations were included, conference papers were excluded; 3) the duration of symptoms was in accordance with the latest definition (≥4 weeks), 4) SRC was the mechanism of injury for all concussed participants or was distinctively analysed as its own sub-type, 5) study population consisted of adults ≥18 years old, 6) symptoms of depression and/or anxiety were screened for and discussed in relation to SRC and PSaC.^[2]^ Google Scholar was included for its extensive cataloguing of literature and powerful search capabilities that complement more traditional search methods.^[23]^ A forward- and backwards-citation search was also performed to identify additional relevant literature by examining the reference lists of included articles and tracking subsequent publications that cited them.

### Study selection and screening

**Fig.1:**
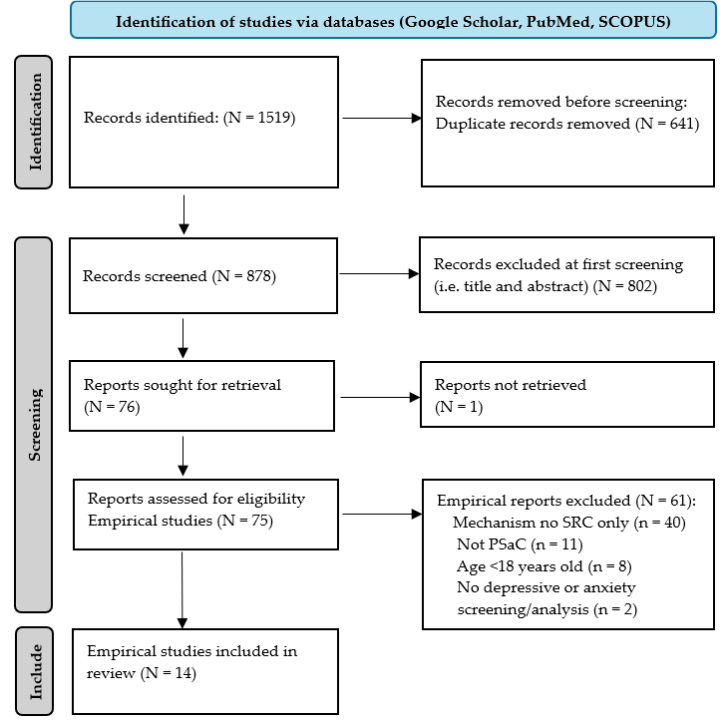
PRISMA Diagram Detailing Scoping Review Process^[25]^

Duplications were removed with the assistance of the automatic tool in Rayyan (Figure 1).^[24]^ The initial screening and eligibility assessment of the articles were conducted by two reviewers (MJL & NS). Disagreements between the reviewers were resolved by consulting the third reviewer (KEW).

### Data extraction

Data extraction was conducted using Microsoft^®^ Excel for Mac (Microsoft Corporation, Version 16.95.3 (25032931). Full text screening was performed by using a data extraction sheet, which was iteratively adjusted to meet the research objectives and answer the research questions.

### Quality appraisal

JBI critical appraisal checklists were used to assign a quality rating for each included article (Supplementary Table 2).^[19]^ A percentage was calculated using the following equation: $\%=\left(\frac{\#\;of\;“\text{Yes}”\;\text{Criteria Selected}}{Total\;number\;of\;applicable\;questions}\right)*100$. Each study was categorised as high (≥85%), moderate (60–84%), or low quality (<60%). Appraisal was performed by the primary reviewer (MJL) and checked by NS. Due to the nature of scoping reviews, studies were not excluded based on their appraisal rating.

Initially the extraction sheet included the article title and variables predominantly relevant for the decision on study inclusion or exclusion: study design (i.e. empirical), number of participants (i.e. extracted SRC participants only), mechanism of injury, age of the concussed participants, time after concussion, whether and how depression and/or anxiety was assessed, and the study’s findings in relation to the association between PSaC and symptoms of depression and/or anxiety.

In line with the exploratory nature of scoping reviews, the data extraction sheet was expanded to include study designs (quantitative observational, mixed methods, qualitative, case study, and experimental designs).

The heterogeneity in study designs and outcomes proved to complicate direct comparison amongst studies. This was addressed by implementing a colour-coding system to determine whether to include, exclude, or contact the authors of a study. Red indicated a clear exclusion of the study, green indicated a clear inclusion, and yellow prompted the reviewers to reach out to the authors for more clarity. An example of a paper receiving a yellow code was not specifically defining the mechanism of injury (i.e. SRC, falls, military, etc.) or not specifically discussing themes surrounding the association between symptoms of depression and/or anxiety and PSaC.

### Data synthesis and confidence rating

The following systematic strategy was implemented to map and compare findings regarding the associations between PSaC, depression, and anxiety: reported associations were classified separately. The main findings reported in each study pertaining to depression and anxiety were also extracted. Predefined criteria were used to categorise each of the variables (i.e. depression and anxiety) direction of association with PSaC (Supplementary Table 3):

1. Positive association: The article found a significant positive association between PSaC and depression/anxiety
2. Inconclusive: Mixed results or insufficient information to determine the direction of the association between depression/anxiety and PSaC.
3. Negative association: The article found a significant inverse association or no significant difference between PSaC and the comparative groups.

A confidence rating of either high, moderate, or low was assigned for each study and displayed in Figure 4. Ratings were assigned by MJL based on the JBI critical appraisal scores, the risk-of-bias rating (Supplementary Table 2), statistical analysis methods, use of validated measurement tools for depression/anxiety, sample size, and the hierarchy of scientific evidence.

Studies were rated with high confidence (i.e. green) when they scored strongly on their JBI critical appraisal tools (≥85%), applied sound statistical methods to analyse data, used valid and reliable tools to screen for depression and anxiety, and whose study designs were higher up on the hierarchy of evidence (i.e. randomised controlled trials).

Moderate confidence (i.e. orange) was applied to studies that presented with adequate JBI scores (60–84%). These studies generally implemented acceptable statistical analysis, however, did not control for confounders, used generally validated screening tools for depression and anxiety, and presented with some methodological limitations. In addition, these studies generally employed study designs that were lower on the hierarchy of evidence (i.e. cross-sectional studies).

Low confidence (i.e. red) was assigned to studies with low JBI appraisal scores (i.e. ≤60%), used non-validated tools for depression and anxiety, presented with basic statistical analysis, and whose study designs were at the lowest level of the hierarchy of scientific evidence (i.e. case reports), thereby limiting the generalisability of the findings.

## Results

The number of study participants, age, time after the concussion and the tools used to capture symptoms of depression and/or anxiety are detailed in Table 1. The specific domains assessed by each screening instrument are detailed in Supplementary Table 4.

**Fig. 2:**
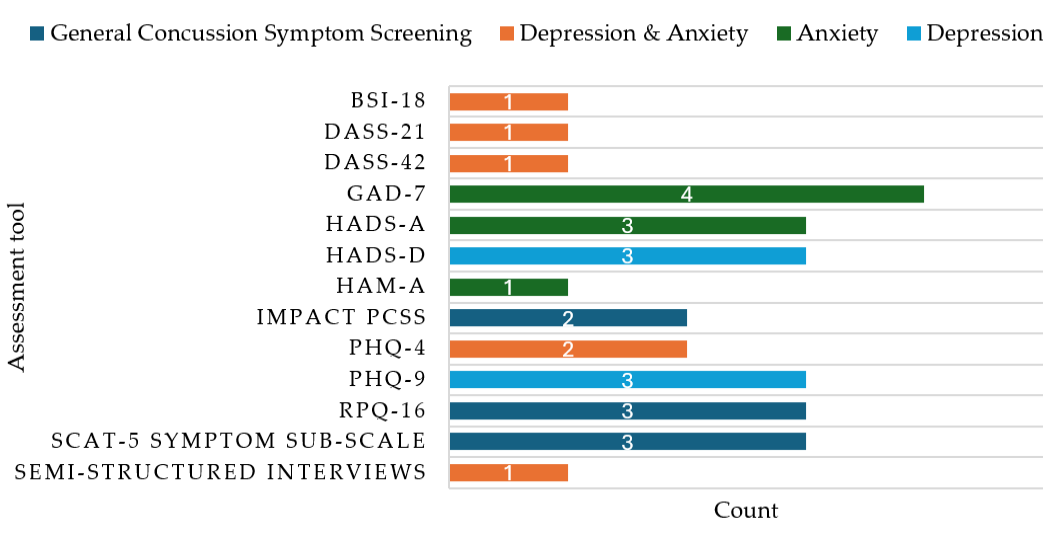
Frequency and purpose of screening tools used in included studies

The frequency count of the tools used in each study to collect data on symptoms of depression and/or anxiety for athletes was charted across four categories: general concussion symptom screening, depressive symptom screening, anxiety symptom screening, and both depression and anxiety symptom screening (Figure 2).

The categories presented in Figure 2 represent differences in psychological constructs assessed across the included studies. Psychological symptom-specific screening instruments (i.e. PHQ-9, GAD-7, HADS, DASS, and BSI-18) are commonly used to screen for symptoms of depression and anxiety, whereas general concussion symptom screening tools (i.e. SCAT-5 Symptom Sub-Scale, ImPACT PCSS, RPQ) include general emotional inventories along with other non-specific symptom burden (i.e. balance, cognitive, sleep, somatic, etc.). These differences make direct comparison across studies challenging and may also be a contributing factor to the reported variability in associations between PSaC and symptoms of depression and/or anxiety.

Eight studies (n = 8, 57%) used depression and anxiety tools in conjunction with post-concussion symptom scales such as the ImPACT Post-concussion Symptom Scale, SCAT-5 Symptom Sub-scale, and the Rivermead Post Concussion Symptoms Questionnaire which include general concussion screening questions.^[11,26,27,29,31,35–37]^ Only one of the included studies relied solely on a post-concussion symptom scale in the form of the SCAT-5 symptom sub-scale.^[32]^ One study utilised only qualitative semi-structured interview techniques to explore themes surrounding depression and anxiety.^[4]^

**Fig. 3:**
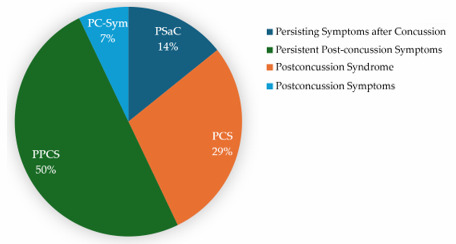
Frequency of terminology used to describe longer lasting concussion symptoms

Terminology used to describe longer lasting symptoms of concussion was extracted and charted (Figure 3).

**Fig. 4:**
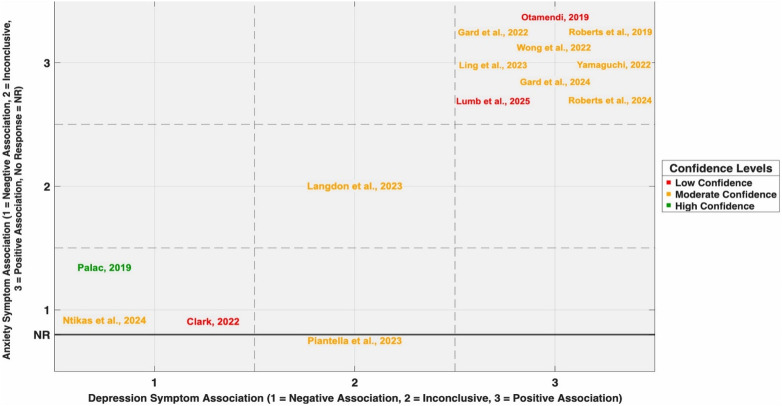
Study matrix showing persisting symptoms after concussion (PSaC) association with symptoms of depression and/or anxiety

In Figure 4 an association matrix displays each study’s concluded associations between PSaC and symptoms of depression and/or anxiety as well as the confidence of each finding (Supplementary Table 3). Out of nine studies (64%) that found positive associations with symptoms of depression and anxiety, two were of low confidence predominantly due to the study designs (i.e. qualitative and case report), while the seven other studies were of moderate confidence generally due to the analytical cross-sectional nature of the study designs, low samples, minimal confounder control, and unadjusted statistical analyses applied. Out of two (14%) studies that returned inconclusive findings, both were of moderate confidence, with one (7%) only reporting on anxiety. Out of three (21%) papers that found negative associations between PSaC and symptoms of depression and anxiety, one was of high confidence due to its randomized control design, one was of moderate quality due to its cohort design and its JBI appraisal score of 73%, and one was of low quality due to it being a low sample case series (i.e. two participants, three cases).

While not a primary objective, the search identified several articles on topics that are beyond this review’s scope, yet they provide useful context for the review’s main findings. Several studies identified factors that may influence symptoms of depression and/or anxiety. These included a history of concussion (especially in females), pre-existing psychiatric conditions, older age, and co-occurring physical symptoms like pain and fatigue.^[30–32]^ A smaller subset of articles discussed interventions.^[29,33,36]^ Their findings suggested that while structured exercise improved physical symptoms, it did not significantly reduce depression and anxiety, indicating a need for specific psychological or pharmacological treatments.^[11,36]^ Finally, two studies explored neurophysiological correlates, linking symptoms of depression and/or anxiety to white brain matter alterations (via magnetic resonance imaging) and specific patterns of brain activity (via quantitative electroencephalogram, qEEG), respectively.^[26,27]^

## Discussion

### Terminology and definitional inconsistency

This scoping review systematically mapped the evidence on symptoms of depression and/or anxiety in athletes experiencing persisting symptoms after a sport-related concussion, identifying three key areas that challenge the field: inconsistent terminology, conflicting findings on the link between PSaC and symptoms of depression and/or anxiety, and a lack of standardised screening tools.

The continued use of inconsistent and varied terminology to describe persisting symptoms after concussion creates a significant challenge for researchers and clinicians, as it complicates literature searches and data comparisons across studies.^[4,11,27–29,31–37]^ This inconsistency also impacts clinical guidelines required for medical practitioners to implement safe and accurate assessments and provide clear communication to athletes who present with PSaC.^[3]^

### Association between PSaC and symptoms of depression and/or anxiety

A primary finding of this review is the association between PSaC and a higher prevalence of depressive and anxiety symptoms that was observed beyond the typical clinical timeframe in which concussion symptoms are expected to resolve.^[2]^ This finding both supports and extends the conclusions of previous work. Across the included studies, depression and anxiety were predominantly assessed and reported together, with only one study examining depression in isolation and none examining anxiety alone (Figure 4). This co-assessment approach precludes a definitive conclusion on whether depression or anxiety was more prevalent in athletes with PSaC. The terms “depression and anxiety” and “depression/anxiety” are used interchangeably throughout this review to reflect this co-assessment pattern in the literature, rather than to imply a single combined diagnostic

For instance, while Lambert et al. (2022) identified a link between persistent symptoms and depression in a mixed population, the current review confirms this association specifically within athlete cohorts, who face unique psychosocial pressures. This association suggests that symptoms of depression and anxiety are not merely an acute reaction to injury but an integral component of the prolonged recovery process, interconnected with physical and cognitive symptoms. This connection is further supported by emerging neurophysiological evidence from the reviewed articles. For instance, studies identifying links between psychological symptoms and objective biomarkers such as white matter alterations or specific qEEG patterns suggest a tangible biological basis for this relationship, moving the discussion beyond purely psychological or behavioural explanations.^[26,27]^

However, the strength of this conclusion is tempered by significant methodological inconsistencies across the included studies. Weaknesses in study design and varied selection of assessment tools likely contribute to the conflicting results found in the literature. This variability is likely compounded by a range of risk factors, such as a history of concussion, pre-existing psychiatric conditions, and athlete age. Studies with different population demographics are likely to yield varied results, highlighting that the relationship between PSaC and symptoms of depression and/or anxiety is not uniform but is moderated by numerous individual variables. This underscores the complexity of the issue, which is further demonstrated by the five studies in this review that reported either inconclusive (n=2) or negative (n=3) associations between PSaC and symptoms of depression and/or anxiety (Figure 4). This variability highlights an ongoing debate and reinforces the need for more robust research to clarify this relationship and inform screening protocols for athletes.^[29,30,32,33,36]^

### Screening tool heterogeneity

This review also reveals a significant lack of consensus on the appropriate tools for screening depression and anxiety in athletes with PSaC, further complicating an already nuanced topic. The included studies employed a wide array of instruments, ranging from general concussion symptom scales (e.g., SCAT-5) to specific measures for depression (e.g., PHQ-9) and anxiety (e.g., GAD-7).

Overall, this heterogeneity presents two major problems. For research, it makes findings difficult to compare across studies and limits confidence in any observed associations. For clinical practice, this lack of uniformity has direct consequences for treatment efficacy. For example, several reviewed articles noted that interventions like structured exercise improved physical symptoms but failed to address symptoms of depression and/or anxiety, which often require targeted therapeutic or pharmacological approaches.^[8,16,26,36,38]^ Without standardised screening to identify these issues, athletes may receive incomplete care, leading to frustration for all parties and complicating the development of clear return-to-play pathways. The findings of this review thus reinforce the call by Iverson et al. (2020) for risk stratification, as the identified methodological weaknesses prevent the development of a clear risk profile for athletes.

### Clinical implications

The findings of this scoping review have implications for clinical practitioners working with SRCs and PSaC. Given that symptoms of depression and anxiety were identified in the majority of included studies as significant presentations of PSaC in athletes, the routine screening and management should be considered an integral component of ongoing concussion management rather than an additional afterthought. This supports the clinical position that psychological and neuropsychological input should be specifically and routinely integrated into the multidisciplinary management protocols for SRCs and PSaC, rather than reserved for referral when symptom presentation does not resolve within the clinically expected timeframe.^[39–41]^ Clinical statements and guidelines have advocated for the formal integration of neuropsychological experts in the evaluation and management of SRC.^[39–41]^ The findings of this review support and advocate for the need for standardised psychological screening, early identification, and targeted intervention as a routine element of athlete care by qualified neuropsychological experts.

Furthermore, the findings highlight the importance of progressing towards standardisation regarding the use of validated screening tools for symptoms of depression and/or anxiety (e.g., PHQ-9, GAD-7) in post-concussion assessment to reduce the risk of overlooking these symptoms.

While methodological improvements in study design, terminology usage, and depression and/or anxiety symptom tool standardisation are viewed as necessary to address inconsistencies in findings related to PSaC, they are not sufficient to address the fundamental clinical and biological differences seen in the condition. Individual variable differences, including the pathological characteristic (i.e. mechanism of injury, number of concussions, burden of disease, etc.), pre-injury psychological status, and individual recovery timelines, may influence the presence and persistence of the discussed symptoms of depression and/or anxiety in this review.

### Limitations and future research

The current scoping review was limited to the English language and, as a result, may have excluded relevant research published in other languages. This may have potentially led to important findings and perspectives being omitted. Future reviews should consider including studies published in different languages to capture a broader, more diverse range of evidence and reduce the risk of language bias.

Several studies examined retired athletic populations that presented with substantial time gaps between initial injury and symptom assessment. These prolonged periods introduce additional confounding factors such as the influence of lifestyle, accumulative health risk exposure, and ageing, which have the potential to complicate associations between PSaC and symptoms of depression and/or anxiety. It is therefore recommended that the findings of this review be interpreted with caution, and that future research aim to address confounding variables, such as time since concussion and match groups by athletic/activity status.

Most studies that used group-based designs treated PSaC as a homogeneous condition. By doing so, individual characteristic differences, pre-injury psychological status, number of previous concussions, and individual prognostic timelines on recovery, to name a few, were not taken into consideration, limiting the interpretability of results and furthermore the application of the findings in a clinical setting. Future research can benefit from defining injury characteristics prior to analysis and conducting subgroup analyses to better capture the heterogeneity in SRC and PSaC presentations. Future researchers are encouraged to build upon the foundations established by this scoping review and conduct a systematic review with meta-analysis, which will allow for more detailed quantitative analysis on the associations between PSaC and symptoms of depression and anxiety.

Furthermore, early and graded exercise prescription is recommended for concussion treatment as per the latest consensus statement. However, guidance on exercise’s role as a prescriptive modality for PSaC is limited, particularly when considering it in relation to symptoms of depression and anxiety. The literature identified in this review was consistent with this, with a lack of exploration on exercise prescription as a modifier for symptoms of depression and anxiety in PSaC.

Stakeholder engagement is a recommended optional step in scoping reviews following the JBI and the Arksey and O’Malley methodology for scoping reviews; however, it was not implemented in this scoping review, as the aim of this review was to map the extent and nature of existing literature rather than attempt to develop guidelines around PSaC and symptoms of depression and/or anxiety.^[18,19,22]^ We, as the reviewers, acknowledge the benefit and value stakeholders can provide in the review process and recommend that this process be implemented in future and may aim to include engagement at a later stage to integrate their viewpoints with the current literature.^[18,19,22]^

Finally, the primary discipline underpinning this scoping review is sports concussion science and sports medicine and as such, a detailed grounding in psychiatric terminology falls outside of the defined scope of this work. A further limitation identified is the heterogeneity in mental health terminology used across the included studies; this review attempts to rectify this by operationalising outcomes as symptoms of depression and/or anxiety measured by validated tools in each study.

Future research should collaborate with psychiatrists and psychologists to more thoroughly explore the psychiatric domains of PSaC, including the development of specific clinical guidelines through methods such as expert consensus or Delphi surveys.

## Conclusion

The collective findings of this scoping review highlight that the associations between PSaC and symptoms of depression and anxiety are reported in most of the included studies with varying direction, strength and confidence of these associations.^[4,11,26–28,31,34,35,37]^ Positive associations were most frequently reported in studies that utilised validated clinical screening tools such as the PHQ-9, GAD-7, HADS, DASS, and BSI-18 scales across both active and retired athletic populations.^[11,26–31,34–37]^ Studies with varying post-injury timeframes, and those that implemented general concussion symptom questionnaires without dedicated psychological symptom measures reported with greater heterogeneity, inconclusive or negative associations.^[29,30,32,33,36,37]^ These findings highlight the need for future research to adopt a definitive definition for persisting symptoms (with PSaC emerging as the preference in recent literature),^[1,3,26]^ utilise standardised depression and anxiety symptom measures, standardise population characteristics and time since injury prior to study inclusion, thus allowing for clearer literature synthesis and data interpretation for future researchers.

## Supplementary Information



## Figures and Tables

**Table 1:** Overview of included studies demographics and tools used to assess symptoms of depression and/or anxiety

Author (year)	Sport participants	Age of concussed participants	Time after concussion	Tools
**Lumb et al. (2025)** ^[26]^	1	21 years old	~13 months	ImPACT PCSS; GAD-7; PHQ-9
**Gard et al. (2024)** ^[27]^	22	27 years (±6.4)	Time from first SRC: 8.9 years (±6.8) Time from last SRC: 2.6 years (±3.2)	SCAT-5 Symptom Sub-Scale; HADS-A; HADS-D
**Roberts et al. (2024)** ^[28]^	296 SRC patients/varsity athletes	20.01 years (±1.69)	Excluded if they had sustained a concussion 6 months before date of testing	GAD-7; PHQ-9; SCAT-5 Symptom Sub-Scale
**Ntikas et al. (2024)** ^[29]^	256 SRC patients/athletes	38.9 years (±18.1)	Main outcomes were explored 6 months post-concussion	GAD-7; PHQ-9; RPQ-16
**Piantella et al. (2023)** ^[30]^	72 SRC patients/professional jockeys	32.79 years (±11.62)	χ̄= 6 years	DASS-21
**Ling et al. (2023)** ^[31]^	560 retired women’s soccer players (320 reported with concussion history)	34 years (±9)	Average time since retirement 12 years (±9 years)	ImPACT PCSS, GAD-7; PHQ-4
**Langdon et al. (2023)** ^[32]^	163 SRC patients	29.2 years (±10.4)	16.7 weeks (±7.7)	SCAT-5 Symptom Sub-Scale
**Wong et al. (2022)** ^[11]^	37 SRC patients (Study 1) 22 SRC patients (Study 3)	χ̄ = 29 years (Study 1) χ̄ = 23.6 years (Study 3)	Male x̃ = 38.5 days (Study 1) Female x̃ = 28 days (Study 1) ≥1 month (Study 3)	DASS-42; RPQ-16 (Study 1 & 3)
**Clark (2022)** ^[33]^	2 SRC patients (Case 1; Case with two sequential concussions: 2a & 2b)	Case 1 = 21 years Case 2a & 2b = 24 years	Case 1 testing done up until 34 days post-concussion Case 2a testing done up until 19 days post-concussion Case 2b testing done up until 23 days post-concussion (second concussion sustained 105 days from first)	HAM-A; BDI
**Yamaguchi (2022)** ^[34]^	13 SRC patients	24.1 years (±5.5)	Testing done at 4 weeks post-concussion	BSI-18
**Gard et al. (2022)** ^[35]^	21 SRC patients/athletes	26 years (±6.5)	Time since last SRC: 2.5 years (±3) Time since first SRC: 9 years (±7)	SCAT-5 Symptom Scale; HADS-A; HADS-D
**Palac et al. (2019)** ^[36]^	34 SRC patients/collegiate athletes	28.79 years (±10.37)	16.38 months (±18.51)	RPQ-16; HADS-A; HADS-D
**Otamendi (2019)** ^[4]^	12 SRC patients/non-elite athletes	χ̄ = 25.9 years	χ̄ = 15.6 months	Semi-structured interviews
**Roberts et al. (2019)** ^[37]^	3406	52.8 years (±14.2)	Last NFL play ranged from 1–55 years prior to testing. x̃ = 24 years IQR = 10–15 years	PHQ-4

BDI, Beck’s Depression Inventory; BSI, Brief Symptom Inventory; DASS-21/42, Depression Anxiety Stress Scale–21/42; GAD-7, General Anxiety Disorder 7-item Scale; HADS-A, Hospital Anxiety and Depression Scale–Anxiety Subscale; HADS-D: Hospital Anxiety and Depression Scale–Depression Subscale; HAM-A, Hamilton Anxiety Rating Scale; ImPACT, Immediate Post-Concussion Assessment and Cognitive Testing; IQR, Interquartile range; mo, Months; NFL, National Football League; PCSS, Post-Concussion Symptom Scale; PHQ-9/4, Patient Health Questionnaire–9/4; PSaC, Persisting Symptoms after Concussion; RPQ, Rivermead Post-Concussion Symptoms Questionnaire; SCAT-5, Sports Concussion Assessment Tool–5th Edition; SRC, Sports-related concussion; χ̄, Mean; x̃, Median; ±, Standard deviation; %, Percent; ~, Approximately

## Data Availability

The study’s raw data and relevant supporting materials will be made available to other researchers upon request. Request for access to the data should be directed to the corresponding author.
