# Supplementary material for: The Association Between Persisting Symptoms after Concussion (PSaC) and Symptoms of Depression and/or Anxiety in the Athletic Population: A Scoping Review
Source: S Afr J Sports Med. 2026 Jun 15;38(1):v38i1a24812. doi: 10.17159/2078-516X/2026/v38i1a24812 (PMC13299698; doi:10.17159/2078-516X/2026/v38i1a24812)
Supplement: Supplementary file 1 [file 2078-516x-38-v38i1a24812-s001.pdf]

# The Association Between Persisting Symptoms after Concussion (PSaC) and Symptoms of Depression and/or Anxiety in the Athletic Population: A Scoping Review

**Supplementary Table 1. Search strings for corresponding databases**

| Database | Search string |
|---|---|
| **Google Scholar** | ("Persistent Concussion Symptoms" OR "Persistent Post Concussion Symptoms" OR "Persistent Post Concussion Syndrome" OR "Persistent Post Concussive Symptoms" OR "Persistent Symptoms after Concussion" OR "Post concussion syndrome") AND (Depression) AND (Anxiety) AND (Sport) |
| **PubMed** | (((((((("Persistent Concussion Symptoms") OR ("Persistent Post Concussion Symptoms")) OR ("Persistent Post Concussion Syndrome")) OR ("Persistent Post Concussive Symptoms")) OR (Persistent Symptoms after Concussion)) OR ("Post concussion syndrome")) AND (Depression)) AND (Anxiety)) AND (Sport*)) AND (2019:2025[pdat]) |
| **SCOPUS** | (TITLE-ABS-KEY("Persistent Concussion Symptoms" OR "Persistent Post Concussion Symptoms" OR "Persistent Post Concussion Syndrome" OR "Persistent Post Concussive Symptoms" OR "Persistent Symptoms after Concussion" OR "Post Concussion Syndrome") AND TITLE-ABS-KEY(Depression) AND TITLE-ABS-KEY(Anxiety) AND TITLE-ABS-KEY(Sport*)) AND PUBYEAR > 2018 AND PUBYEAR 2025 AND PUBYEAR > 2018 AND PUBYEAR < 2025 AND ( LIMIT-TO ( DOCTYPE,"ar" ) OR LIMIT-TO ( DOCTYPE,"re" ) ) |

*TITLE-ABS-KEY, Search restricted to title, abstract, and keywords (Scopus syntax); pdat, Publication date field (PubMed syntax); PUBYEAR, Publication year (Scopus syntax); LIMIT-TO (DOCTYPE,”ar”), Restricts to articles; LIMIT-TO (DOCTYPE,”re”), Restricts to reviews; *,Wildcard to capture word variations; Boolean operators AND / OR were used to combine search terms. Date limits (2019–2025) applied to include studies published from January 2019 to February 2025*

**Supplementary Table 2. Joanna Briggs Institute critical appraisals of included studies**

| Author (year) | Study aim | JBI critical appraisal tool/study design | Score (%) | Quality rating | Design-based-risk-of-bias |
|---|---|---|---|---|---|
| Lumb et al. (2025)[26] | To provide a replicable framework for clinicians to monitor a patient's recovery progress in terms of brainwave activity, general cognition, symptoms of depression and anxiety and motor control PSaC | Case report | 100 | High | High |
| Gard et al. (2024)[27] | To assess whether widespread white brain matter in concussed athletes with PPCS differed from a control using 7T diffusion magnetic resonance imaging | Analytical cross-sectional study | 100 | High | Moderate |
| Roberts et al. (2024)[28] | Explore the associations between SCAT-5 symptoms reporting and gold standard measures for anxiety and depression as well as explore the use of the SCAT-5 symptoms subscale to identify anxiety and depression symptomology in post-concussion athletes | Analytical cross-sectional study | 75 | Moderate | Moderate |
| Ntikas et al. (2024)[29] | To compare the outcomes, including mental health, of individuals reporting with SRC & post-concussion symptoms to those reporting with non SRC traumatic brain injury (TBI) | Cohort study | 73 | Moderate | Moderate |
| Piantella et al. (2023)[30] | To concurrently assess the potential interconnection between concussion history, workplace stress, and depression symptoms in professional female jockeys | Analytical cross-sectional study | 88 | High | Moderate |
| Ling et al. (2023)[31] | To investigate the risks and benefits of elite women's soccer careers in general, musculoskeletal, reproductive, post-concussion symptoms, and mental health domains. | Analytical cross-sectional study | 88 | High | Moderate |
| Langdon et al. (2023)[32] | To explore the heterogeneity of Persistent symptoms after SRC | Analytical cross-sectional study | 88 | High | Moderate |
| Wong et al. (2022)[11] | Aim 1: To compare adult athletes with prolonged symptoms after SRC to a healthy control exploring concussion symptoms, mental health, fear, sleep and disability<br>Aim 2: N/A<br>Aim 3: Explore and understand psychological and sociological impacts of prolonged symptoms after SRC in adult athletes and explore deeper insights to the athletes lived experiences | Aim 1: Analytical cross-sectional study<br>Aim 2: N/A<br>Aim 3: Mixed Methods (i.e. Analytical cross-sectional study & Qualitative study) | Aim1: 100<br>Aim 2: N/A<br>Aim 3: 80 | Aim 1: High<br>Aim 2: N/A<br>Aim 3: Moderate | Moderate |

*Quality ratings: High (≥85%); Moderate (60–84%); Low (<60%). JBI, Joanna Briggs Institute; N/A, Not applicable; PCS, Post-concussion syndrome; PPCS, Persistent post-concussion symptoms; PSaC, Persisting Symptoms after Concussion; SRC, Sports-related concussion; TBI, Traumatic brain injury; %, Percent. Date limits (2019–2025) applied to include studies published from January 2019 to February 2025*

**Supplementary Table 2. Continued. Joanna Briggs Institute critical appraisals of included studies**

| Author (year) | Study aim | JBI critical appraisal tool/study design | Score (%) | Quality rating | Design-based-risk-of-bias |
|---|---|---|---|---|---|
| Clark (2022)[33] | To track student athletes' mental health throughout the season and post-concussion | Case Series | 90 | High | High |
| Yamaguchi (2022)[34] | Aim 1: N/A<br>Aim 2: N/A<br>Aim 3: To examine if athletes with PPCS displayed greater depression, anxiety, and sensory reweighting impairments in postural control compared to a matched health control<br>Aim 4: N/A | Aim 1: N/A<br>Aim 2: N/A<br>Aim 3: Analytical cross-sectional study<br>Aim 4: N/A | Aim 1: N/A<br>Aim 2: N.A<br>Aim 3: 75<br>Aim 4: N/A | Aim 1: N/A<br>Aim 2: N/A<br>Aim 3: Moderate<br>Aim 4: N/A | Moderate |
| Gard et al. (2022)[35] | Identify the cause of vestibular impairment in athletes with PPCS. | Analytical cross-sectional study | 100 | High | Moderate |
| Palac et al. (2019)[36] | To examine the efficacy of supervised vs home-based exercise programs in treating individuals who have PCS | Randomized control trial | 69 | Moderate | Low |
| Otamendi (2019)[4] | To thematically represent PPCS athlete's experiences, appraisals and coping strategies | Qualitative study | 80 | Moderate | High |
| Roberts et al. (2019)[37] | To determine the impact that duration of play, position, and experience of concussions impact on athletes' cognition-related quality of life, depression and anxiety | Analytical cross-sectional study | 100 | High | Moderate |

*Quality ratings: High (≥85%); Moderate (60–84%); Low (<60%). JBI, Joanna Briggs Institute; N/A, Not applicable; PCS, Post-concussion syndrome; PPCS, Persistent post-concussion symptoms; PSaC, Persisting Symptoms after Concussion; SRC, Sports-related concussion; TBI, Traumatic brain injury; %, Percent. Date limits (2019–2025) applied to include studies published from January 2019 to February 2025*

**Supplementary Table 3. The association between PSaC and symptoms of depression and/or anxiety**

| Author (year) | Depression symptom association | Anxiety symptom association | Confidence of findings | Reasoning for confidence rating |
|---|---|---|---|---|
| Lumb et al. (2025)[26] | Positive association | Positive association | Low | Single case report; PHQ-9 (5) and GAD-7 (5) both indicate mild symptoms; Positive association noted in discussion; unadjusted |
| Gard et al. (2024)[27] | Positive association | Positive association | Moderate | HADS-D and HADS-A scores were significantly higher in the SRC group vs matched controls ($p < 0.001$); unadjusted analysis |
| Roberts et al. (2024)[28] | Positive association | Positive association | Moderate | SCAT-5 symptoms of feeling anxious, feeling more emotional, irritability, and sadness had the strongest correlations with the GAD-7 ($r > 0.400$; $p < 0.001$); Symptoms of concentration problems, fatigue, feeling anxious, feeling slowed down, irritability, memory problems, mental fog, sadness and trouble falling asleep had the highest correlations with the PHQ-9 ($r > 0 .400$; $p < 0.001$) |
| Ntikas et al. (2024)[29] | Negative association | Negative association | Moderate | Significantly lower prevalence of depression in the SRC group was present compared to Non-SRC group at 6 mo post-concussion ($p = 0.01$)<br>Significantly lower prevalence of Anxiety in the SRC group was present compared to Non SRC group at 3 mo ($p = 0.05$) and at 6 mo post-concussion (0.03)<br>Adjusted analysis for clinical and demographic variables were implemented using binary logistic regression |
| Piantella et al. (2023)[30] | Inconclusive | NR | Moderate | The pathological group had higher negative symptoms of depression than the control. The association was non-significant after controlling for age, gender, and workplace stress. Anxiety not tested |
| Ling et al. (2023)[31] | Positive association | Positive association | Moderate | A dose response was present with fewer mental health symptoms presenting the longer the athlete had been retired with the most recently retired groups (0-5 and 6-10 years) displaying higher severity of depressive and anxiety symptoms. An ANOVA showed significant differences across all retirement categories (PHQ-4: $p = 0.001$; GAD-7: $p =0.004$). Symptoms were self-reported and unadjusted |
| Langdon et al. (2023)[32] | Inconclusive | Inconclusive | Moderate | Only used SCAT-5 symptom scale to assess symptoms and identify subtypes of PSaC. Migraine-emotional ($p < 0.001$, $d = 0.82$) and neurocognitive-emotional ($p < 0.001$, $d = 0.71$) subgroups displayed significantly higher emotional symptom scores compared to the others. No use of more sensitive scales to assess depression and/or anxiety |

*Associations: 1 = Negative association, 2 = Inconclusive, 3 = Positive association; Confidence of Findings: 1 = Low, 2 = Moderate, 3 = High. ANOVA, Analysis of variance; BDI, Beck's Depression Inventory; BSI-18, Brief Symptom Inventory (18-item); DASS-42, Depression Anxiety Stress Scale–42; d, Cohen's d effect size; GAD-7, General Anxiety Disorder 7-item Scale; HADS-A, Hospital Anxiety and Depression Scale–Anxiety Subscale; HADS-D, Hospital Anxiety and Depression Scale–Depression Subscale; MANOVA, Multivariate analysis of variance; mo, Months; NR Not reported; p, Probability value; PHQ-9, Patient Health Questionnaire–9; PSaC, Persisting Symptoms after Concussion; PCS: Post-concussion Syndrome; RPQ, Rivermead Post-Concussion Symptoms Questionnaire; r, Pearson correlation coefficient; SCAT-5, Sports Concussion Assessment Tool–5th Edition; SRC, Sports-related concussion*

**Supplementary Table 3. Continued. The association between PSaC and symptoms of depression and/or anxiety**

| Author (year) | Depression symptom association | Anxiety symptom association | Confidence of findings | Reasoning for confidence rating |
|---|---|---|---|---|
| Wong et al. (2022)[11] | Positive association | Positive association | Moderate | DASS-42 and RPQ (split into RPQ3 and 13) used. Mild depression and moderate anxiety was present in the SRC group when compared to control. Significance was present for both after performing a MANOVA ($p = 0.001$). Anxiety correlated with stress ($r = 0.7$) and depression ($r = 0.61$). Depression correlated with RPQ13 ($r = 0.60$) |
| Clark (2022)[33] | Negative association | Negative association | Low | Only two participants (one with two concussions). Only meeting PSaC definition for Case 1. No control for confounders and high individual variability. Case 1 post-concussion depression and anxiety scores were clear at 34 days (i.e. HAM-A = 0; BDI = 0). Case 2a post-concussion depression and anxiety scores were clear at day 19 (i.e. HAM-A = 0; BDI = 0). Case 2b post-concussion anxiety scores were clear at day 23 (i.e. HAM-A = 0) and a score of 1 for depression was reported (BDI = 1) |
| Yamaguchi (2022)[34] | Positive association | Positive association | Moderate | The post-concussion group scored significantly higher than the control group on the BSI-18 depression and anxiety subscales ($p < 0.05$). Relatively small sample size that relied on self-reported measures |
| Gard et al. (2022)[35] | Positive association | Positive association | Moderate | HADS-A and HADS-D scores were significantly higher ($p < 0.001$) in the PPCS group compared to matched control. Strong statistical evidence present |
| Palac et al. (2019)[36] | Negative association | Negative association | High | Aerobic exercise (with or without cognitive training) in athletes with prolonged symptoms after concussion associated symptoms, mental fatigue, and improved mindful attention and mobility, but had no significant effect on HADS-D or HADS-A scores. Possible reasoning could include intervention not being targeted for specifically psychological disorders, insufficient dose, and need for ongoing psychological treatment |
| Otamendi (2019)[4] | Positive association | Positive association | Low | Study reported themes of sadness, withdrawal and fear of activity. SCAT-5 Symptom Sub-Scale results included 7/12 participants scoring on nervous/anxious and 6/12 scoring on sadness |
| Roberts et al. (2019)[37] | Positive association | Positive association | Moderate | Strong correlation with SCAT-5 Symptom Sub-Scale variables (i.e. anxious, sadness, irritability, more emotional) with GAD-7 (r's > 0.400, p's < 0.001). Strong correlation with SCAT-5 Symptom Sub-Scale variables (i.e. sadness, trouble sleeping, concentration problems, slowed down, anxious, irritability, mental fog, fatigue, memory problems) with PHQ-9 (r's > 0.400, p's < 0.001). Emotional sub-scale of SCAT-5 predicted mild to severe anxiety on the GAD-7 ($p < 0.001$). The Sleep, Cognitive, and Emotional subscales predicted mild to severe depression on the PHQ-9 (p's < 0.05) |

*Associations: 1 = Negative association, 2 = Inconclusive, 3 = Positive association; Confidence of Findings: 1 = Low, 2 = Moderate, 3 = High. ANOVA, Analysis of variance; BDI, Beck's Depression Inventory; BSI-18, Brief Symptom Inventory (18-item); DASS-42, Depression Anxiety Stress Scale–42; d, Cohen's d effect size; GAD-7, General Anxiety Disorder 7-item Scale; HADS-A, Hospital Anxiety and Depression Scale–Anxiety Subscale; HADS-D, Hospital Anxiety and Depression Scale–Depression Subscale; MANOVA, Multivariate analysis of variance; mo, Months; NR Not reported; p, Probability value; PHQ-9, Patient Health Questionnaire–9; PSaC, Persisting Symptoms after Concussion; PCS: Post-concussion Syndrome; RPQ, Rivermead Post-Concussion Symptoms Questionnaire; r, Pearson correlation coefficient; SCAT-5, Sports Concussion Assessment Tool–5th Edition; SRC, Sports-related concussion*

**Supplementary Table 4. Psychological symptom domains assessed by symptoms of depression and/or anxiety and concussion screening tools**

| Instrument | Primary symptoms assessed |
| --- | --- |
| **PHQ-9** | Depressive symptoms severity |
| **PHQ-4** | Combined depressive symptoms severity and anxiety symptoms screening |
| **GAD-7** | Anxiety symptoms screening |
| **HADS-A** | Anxiety symptoms screening |
| **HADS-D** | Depressive symptom screening |
| **DASS-21** | Depressive symptoms, anxiety symptoms, and stress symptoms screening (short form) |
| **DASS-42** | Depressive symptoms, anxiety symptoms, and stress symptoms screening (full form) |
| **BSI-18** | Psychological distress symptoms (PDS) with depression and anxiety symptom sub-scales |
| **HAM-A** | Anxiety symptoms screening |
| **BDI** | Depressive symptoms severity screening |
| **SCAT-5 Symptom Sub-Scale** | General concussion symptom screening including emotional screening items |
| **ImPACT PCSS** | General post-concussion symptom screening including emotional screening items |
| **RPQ-16** | General post-concussion symptom screening including emotional screening items |
| **Semi-structured Interviews** | Qualitative exploration of emotional and psychological experiences due to concussion |

*BDI, Beck Depression Inventory; BSI-18, Brief Symptom Inventory (18-item); DASS-21/42, Depression Anxiety Stress Scales (21- and 42-item versions); GAD-7, Generalized Anxiety Disorder 7-item scale; HADS-A/D, Hospital Anxiety and Depression Scale – Anxiety/Depression subscales; HAM-A, Hamilton Anxiety Rating Scale; ImPACT PCSS, Immediate Post-Concussion Assessment and Cognitive Testing – Post-Concussion Symptom Scale; PHQ-4/9, Patient Health Questionnaire (4- and 9-item versions); RPQ-16, Rivermead Post-Concussion Symptoms Questionnaire (16-item); SCAT-5, Sports Concussion Assessment Tool – 5th Edition symptom sub-scale*
